# Hyperactivity in anorexia nervosa: to warm or not to warm. That is the question (a translational research one)

**DOI:** 10.1186/s40337-018-0190-6

**Published:** 2018-03-02

**Authors:** Olaia Carrera, Emilio Gutiérrez

**Affiliations:** 1 Unidad Venres Clínicos, Facultad de Psicología, Campus Vida, Universidad de Santiago, 15782 Santiago de Compostela, Spain; 2 Departamento de Psicología Clínica y Psicobiología, Facultad de Psicología, Universidad de Santiago, Campus Vida, 15782 Santiago de Compostela, Spain

**Keywords:** Heat treatment, Overexercise, Activity-based anorexia

## Abstract

In the Editorial ‘Is the neglect of exercise in anorexia nervosa research a case of “running out” of ideas or do we need to take a “LEAP” of faith into the future?’ these authors express their doubts concerning the suitability of keeping patients warm as a beneficial treatment option in managing excessive activity in anorexia nervosa (AN) patients. The case for warming as an adjunctive treatment for AN patients is based on strong experimental evidence gathered from research on animals with Activity-Based Anorexia (ABA). We posit that the beneficial effect of heat results, at least in part, from heat blocking the vicious cycle that hyperactivity plays on AN. Hyperactivity decreases caloric intake by interfering with feeding and increases energy expenditure through excess motor activity which in turn increases emaciation that further strengthens anorexic thinking.

## ᅟ

In number 5 of this journal two of its Editors-in-Chief [1] wrote an extremely important editorial underscoring how excessive activity has been traditionally overlooked in AN research and treatment. In their editorial Touyz and colleagues congratulate themselves for the air-conditioned system in their hospital wards that allowed inpatients with AN to be safe from one of the hottest heat waves ever recorded in Sydney (around 40 °C). The reasoning was that if these patients had not been protected by the air conditioning system their appetite characterized by a restrictive eating pattern would have diminished further as “increasing ambient temperature may in fact contribute towards decreased appetite” [1].

The exaltation of the rescuing properties of the air conditioning system was used as an argument against the advice of Carrera and colleagues [2] that “keeping patients warm may prove to be a beneficial treatment option” in reducing overexercise. Furthermore, Touyz and colleagues recognize that this advice was consistent with research on an analogous animal model of the AN disorder known as activity-based anorexia (ABA), which has experimentally demonstrated that ambient temperature (AT) is a critical factor contributing to the expression of excessive running activity in ABA [3], and that increasing AT to 32 ° C completely prevented and fully reversed excessive activity in ABA rats. In spite of these findings, Touyz and colleagues have warned against Carrera and colleagues’ advice invoking the Hippocratic maxim “primum non nocere”.

We fully accept the guiding Hippocratic maxim in developing new treatment strategies for AN treatment, and we also agree with the mounting evidence that elevated AT decreases food intake in healthy, normal body weight people [4]. However, we stress that Touyz and colleagues’ assumptions that a) elevated AT would have the same decreasing effect in the appetite of underweight AN patients, and b) that air-conditioned ward temperature facilitates patients’ meal consumption, are just unproven assumptions. As far as we are aware, these assumptions are unfounded and unjustified and they should also be seen through the lens of the aforementioned Hippocratic principle.

Interestingly, the suggestion of keeping patients warm is not new, and supplying AN patients with heat was first recommended by William Gull [5]. Nevertheless, in spite of the influential role of Gull, the application of heat to AN patients was almost entirely neglected since the turn of the 20th century. One of the last mentions of the use of heat in the 19th century appeared in the first documented necropsy of a patient who died of self-starvation, which noted that all efforts were made to maintain her warm even by wrapping the patient with bandages [6]. In the 20th century references to warming AN patients were scarce. In 1931, a report [7] informed of a treatment of a series of 20 cases of functional anorexia treated at Ruthin Castle, a private hospital for the investigation and treatment of obscure medical diseases, where patients were kept in bed in a warm room (although the recommended room temperature was unusually low, 60 ° F, for today’s standards). Likewise, in the same decade, a German report [8] mentioned using heating pads and an electric blanket for treating a 17-year old girl.

Since then, to our knowledge, there have been no reports until the beginning of the 21st century with the reported outcomes of three cases treated with three different heat treatment strategies: continuous exposure to a warm environment, intermittent use of a thermal waistcoat, and intermittent sauna baths in an infrared enclosure [9]. These patients showed a reduction in hyperactivity, anxiety, depression which antedated body weight recovery. However, in this case report there was no control group and compliance could not be assessed because the study was conducted on outpatients. A randomized controlled trial of warming in AN was published in 2004, where anorexia nervosa diagnosed patients were warmed with the intermittent use of a thermal waistcoat for three weeks, showed no change in the rate of weight gain; nevertheless, patients wearing thermal waistcoats asked to continue using them and a reduction in parotid gland size was observed [10]. Moreover, in 2002, a randomized, controlled trial of a novel treatment named Mandometer, recommending a treatment package of warming and restricted physical activity, reported a dramatic benefit in weight recovery with a low level of relapse in a mixed sample of patients diagnosed with anorexia nervosa and bulimia nervosa [11]. Furthermore, these findings were corroborated by the results of the multi-site analyses of outcomes after a median of 12.5 months of treatment [12]. However, another randomized controlled trial reported the Mandometer treatment was not superior to treatment as usual for anorexia nervosa [13].

It is interesting to note that as was the case with Gull’s recommendation, the current renewed interest in warming is based on the evidence accrued from starved animals. It is also worth noting that the reputed researcher Prof. Peter Beumont, who firmly maintained that hyperactivity is a relevant manifestation of AN, considered ABA to be a relevant analogous model of AN [14, 15]. ABA stands as the best animal model of the main signs of the disorder (weight loss, reduced food intake, excessive physical activity, hypothermia, disturbed sleep, and alterations in various neuroendocrine axes such as the hypothalamic-pituitary-adrenal/ gonadal axis, as well as alterations in diverse appetite-regulating hormones, and severe grey and white matter volume reductions).

Recent research has established the crucial role of ambient temperature (AT) in reversing exhaustive running activity, severe weight loss, and self-starvation in rats simultaneously placed on a restricted feeding schedule and given free access to an activity wheel [3]. The manipulation of AT in ABA rats has ended the period of neglect of AT which had hitherto deprived us from recognizing the pivotal role of AT in the fate of rats exposed to ABA [16]. Although the results of animal studies are neither a necessary nor sufficient condition for extrapolation to AN patients, we should not underestimate their value or incur in certain errors occurring in the animal laboratory regarding environmental temperature. In other words, inadequate control of ambient temperature has been a common flaw in animal research [17], where: “Historically, the profound impact of ambient temperature on the thermoregulatory capacity of mice and rats has been ignored, with the choice of housing ambient temperature commonly dictated in terms of the comfort of laboratory personnel, typically 20 – 22° C”. (p. 275).

In this sense, with respect to the management of hypothermia, a sign present in AN patients, research members of the Minnesota semi-starvation study underscore the pertinence of “warm clothing, warm blankets, and some warm place where people can spend their daytime hours” [18]. Thus, in their booklet for personnel working with war-starved populations, staff are warned about the failure to consider the influence of ambient temperature and protection from the cold, advancing that bad weather was a sufficient cause to explain the frequent irritability and mood swings of starved people as: “warm, sunny days brightened the spirits immeasurably, while cold, damp, cloudy days lowered the men further in their abyss of dejection”. Notwithstanding, the role of AT and climate on the course of AN has been unduly overlooked in research.

Touyz and colleagues stated that enhanced cognitive behaviour therapy (CBT-E) is promising in addressing unhealthy exercise in adults with AN, and they are also convinced of the promising effects of a semi-structured problem-oriented intervention, compuLsive Exercise Activity theraPy (LEAP) developed with factors that maintain compulsive exercise behaviour at its essence. However, while we eagerly await the final publication of these promising results we are aware of the main problems implicit in establishing the efficacy, effectiveness and efficiency of any psychological treatment is to not get caught up in an endless game. Far from the possibility of a crucial experiment, the foundations that support psychological treatments are immune to any possibility of refutation. In addition to the associated problems of manualization, adherence and competence to deliver a particular treatment, there is the associated problem of allegiance in the context of research on treatment outcome.

However, the parameters involved in supplying heat to AN patients to help them cope with hyperactivity is unquestionably a more objective treatment easy to implement in terms of timing and temperature degrees. A recent example of treatment translation from ABA is the report of the anxiolytic effect of warmth in anorexia nervosa [19]. In this study postprandial anxiety was significantly reduced in patients resting for half an hour immediately after lunch in a room at 32 °C, the same AT which reverses ABA [3]. Given the high level of pre-meal anxiety characteristic of AN patients, the significant decrease in postprandial anxiety is far greater than that achieved by conventional treatments in patients with comparable levels of pre-meal anxiety such as Exposure and Response Prevention [20].

Furthermore, ABA research suggests that excessive exercising in AN patients might somehow be viewed as playing a thermoregulatory function. Consequently, hypothermia provides a new perspective to the vicious cycle between hyperactivity and food refusal, two salient characteristics of anorexia nervosa. From this perspective, excessive exercising in AN is seen as a bio-behavioural response to remedy hypothermia, rather than being driven exclusively by psychological factors such as excessive concern with body weight and shape [21].

## Conclusion

Going back again to Hippocrates, there is also an interesting quote warning us against overlooking AT: “*Whoever wishes to pursue properly the science of medicine must proceed thus. First he ought to consider what effects each season of the year can produce; for the seasons are not at all alike, but differ widely both in themselves and at their changes*”. The possibility exists, beyond reasonable doubt, that AT has been traditionally overlooked in AN research and that neglect has impeded progress of a better understanding of the possibilities of the use of heat in the treatment of AN. We agree with Touyz and colleagues that hyperactivity is the Cinderella of AN signs and as in the case of Cinderella, rodents come to the rescue of hyperactivity to occupy its rightful place in the DSM Court.
